# Dendrimeric HIV-peptide delivery nanosystem affects lipid membranes structure

**DOI:** 10.1038/s41598-021-96194-x

**Published:** 2021-08-19

**Authors:** Katarzyna Milowska, Aleksandra Rodacka, Sophie Melikishvili, Adam Buczkowski, Bartlomiej Pałecz, Iveta Waczulikova, Tibor Hianik, Jean Pierre Majoral, Maksim Ionov, Maria Bryszewska

**Affiliations:** 1 Department of General Biophysics, Faculty of Biology and Environmental Protection, University of Lodz, Pomorska 141/143, 90-236 Lodz, Poland; 2 Department of Molecular Biophysics, Faculty of Biology and Environmental Protection, University of Lodz, Pomorska 141/143, 90-236 Lodz, Poland; 3 Faculty of Mathematics, Physics and Informatics, Comenius University, Mlynska dolina, 842 48 Bratislava, Slovakia; 4 Unit of Biophysical Chemistry, Department of Physical Chemistry, Faculty of Chemistry, 165 Pomorska St., 90-236, University of Lodz, Lodz, Poland; 5 Laboratoire de Chimie de Coordination du CNRS (LCC), 205 Route de Narbonne, 31077 Toulouse Cedex 4, France

**Keywords:** Biological techniques, Biophysics, Biotechnology, Drug discovery

## Abstract

The aim of this study was to evaluate the nature and mechanisms of interaction between HIV peptide/dendrimer complexes (dendriplex) and artificial lipid membranes, such as large unilayered vesicles (LUV) and lipid monolayers in the air–water interface. Dendriplexes were combined as one of three HIV-derived peptides (Gp160, P24 and Nef) and one of two cationic phosphorus dendrimers (CPD-G3 and CPD-G4). LUVs were formed of 1,2-dimyristoyl-sn-glycero-3-phosphatidylcholine (DMPC) or of a mixture of DMPC and dipalmitoyl-phosphatidylglycerol (DPPG). Interactions between dendriplexes and vesicles were characterized by dynamic light scattering (DLS), fluorescence anisotropy, differential scanning calorimetry (DSC) and Langmuir–Blodgett methods. The morphology of formed systems was examined by transmission electron microscopy (TEM). The results suggest that dendriplexes interact with both hydrophobic and hydrophilic regions of lipid bilayers. The interactions between dendriplexes and negatively charged lipids (DMPC–DPPG) were stronger than those between dendriplexes and liposomes composed of zwitterionic lipids (DMPC). The former were primarily of electrostatic nature due to the positive charge of dendriplexes and the negative charge of the membrane, whereas the latter can be attributed to disturbances in the hydrophobic domain of the membrane. Obtained results provide new information about mechanisms of interaction between lipid membranes and nanocomplexes formed with HIV-derived peptides and phosphorus dendrimers. These data could be important for the choosing the appropriate antigen delivery vehicle in the new vaccines against HIV infection.

## Introduction

Human immunodeficiency virus (HIV) is a retrovirus that causes acquired immunodeficiency syndrome (AIDS). HIV-1 is the most prevalent cause of HIV infection worldwide. In its structure, the virus contains a core with a single-stranded RNA and proteins, a protein capsid and a lipid envelope^1^. HIV attacks and destroys T helper lymphocytes (T-cells), which are crucial for the immune system and its responsiveness^2^. AIDS remains a serious problem, although since the mid-90s antiretroviral therapy (ART) has been available. This therapy significantly improves the quality of life for HIV-infected persons, but there are restrictions on its use. Therefore, some research is focused on developing of new preventive and therapeutic vaccines, and on ways of successful delivery of them to target cells^3,4^.

A promising approach to improving the deteriorated immune function in HIV-1-infected individuals may be the application of dendritic cells (DCs) as a vaccine adjuvant. Due to their immune stimulatory potential, dendritic cells have been explored as cellular vaccines or adjuvant in immunotherapies against cancer and viral infections, such as HIV infection^5–8^. Monocyte-derived DC (mo-DC) pulsed ex vivo with viral or tumoral antigens induce a potent protective immune response^6,9,10^.

For therapeutic purposes, the antigen (HIV-derived peptide) needs to be transported through the cell membrane into the cytoplasm of DCs. However, HIV peptides are polyanions that do not penetrate easily into the cells, and therefore require an efficient delivery system. We recently studied the interaction of HIV-derived peptides contained dendriplexes based on carbosilane dendrimers (CBD) with LUVs and lipid monolayers^11^. Two types of CBD composed of carbon–silicon or carbon–oxygen bonds in their core were used; both types of dendrimers contained 16 positive charges at their surface. Using fluorescence anisotropy, dynamic light scattering, Doppler laser velocimetry, Langmuir–Blodgett technique and transmission electron microscopy, we found that both types of dendriplexes interact with model membranes, resulting in increases of fluorescence anisotropy, liposome size and surface pressure. There was an increase of zeta potential from negative to positive values, especially for negatively charged vesicles composed of DMPC–DPPG (9:1 mol/mol).

Other perspective drug carriers can be designed based on cationic phosphorus dendrimers (**CPD**). **CPD** possess a hydrophilic surface and a hydrophobic backbone, which allows for efficient membrane penetration^12^. Because of water-solubility, most of the potential applications of phosphorus-containing dendrimers are related to biology and medicine. Phosphorus dendrimers have some anti-inflammatory properties^13^, and can stimulate NK cell proliferation^14^. Earlier research showed that polycationic phosphorus dendrimers could be used in therapies against HIV infection^15^, as transporters of drugs and gene material^12,16^, and can prevent the aggregation of proteins associated with neurodegenerative processes, e.g. prions^17^, Alzheimer's and Parkinson's diseases^18–21^.

In this paper we have focused on dendriplexes formed by **CPD** of third (G3) and fourth (G4) generation that contain 48 or 96 positive charges at their surface, respectively, and HIV-derived peptides. Gp160, P24 and Nef peptides were chosen due their ability to activate an efficient and strong anti-HIV immune response and to be common for the most HIV strains. Moreover these peptides can be complexed with dendrimers^3^ and delivered to the cells as part of peptide/dendrimer complex.

The aim of the current study was to assess the nature and mechanism of interaction of the HIV peptide/phosphorus dendrimer complex with artificial membranes. Interactions between dendriplexes and vesicles were characterized by dynamic light scattering, fluorescence anisotropy and Langmuir–Blodgett techniques. The morphology of formed systems was examined by transmission electron microscopy (TEM). The interaction of phosphorus dendrimer/HIV-peptide complexes with lipid membrane matrix have never been studied before. Moreover the applying of DSC technique allowed to analyze the membrane thermodynamic parameters at the presence of nanocomplexes.

**Figure 1:**
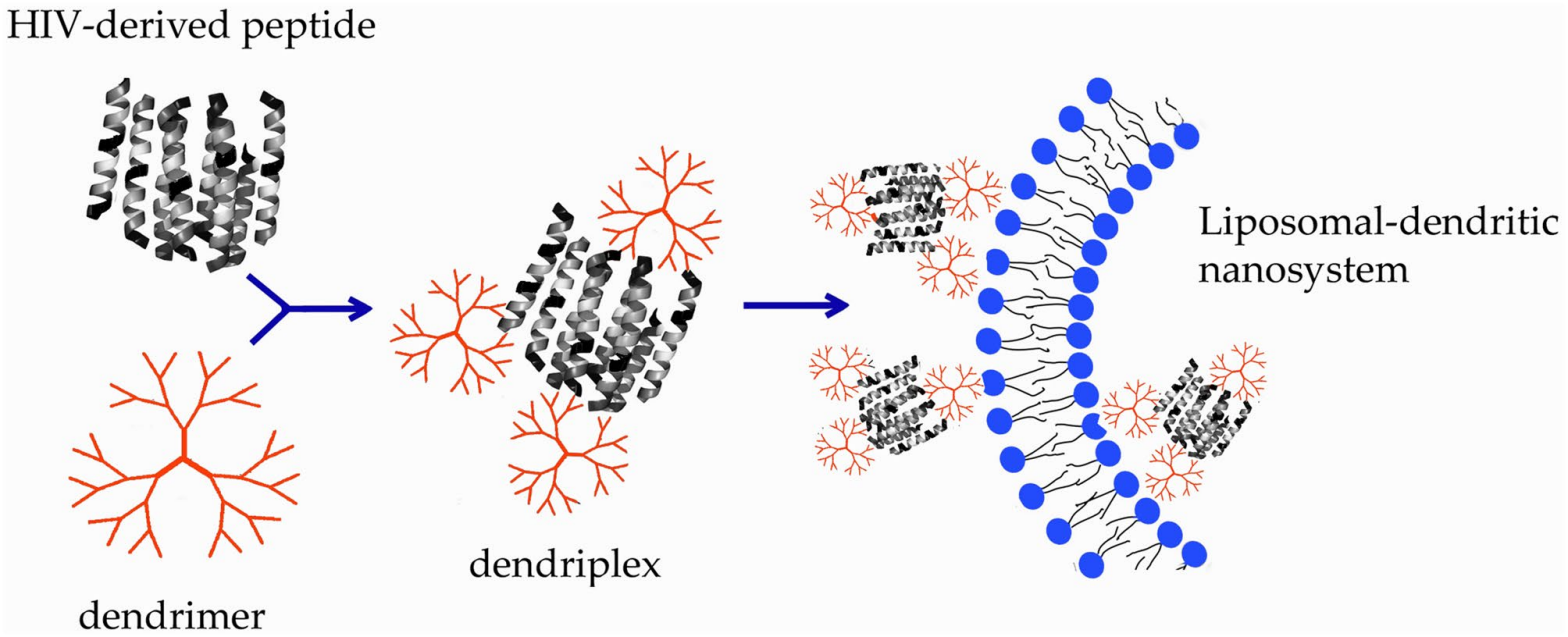
Schematic presentation of the interaction of peptide/dendrimer complexes and liposomal membrane.

How these dendriplexes interact with model lipid membranes was explored, using large unilamellar vesicles (LUV) and lipid monolayers composed of dimyristoylphosphatidylcholine (DMPC) and dipalmitoylphosphatidylglycerol (DPPG). The schematic presentation of the described kind of interactions is shown in Fig. 1. This should broaden our understanding of the mechanisms of the interaction between HIV peptides complexed with phosphorus dendrimers and lipid membranes, which constitute a model of the dendritic cell surface. This might lead to a new strategy for immunotherapy of HIV-1 infection using dendritic cells loaded with synthetic HIV-derived peptides.

## Results and discussion

### Average size and surface charge

In order to model the interaction between HIV peptide/dendrimer complexes (dendriplexes) with a cell membrane, the liposomes of different lipid composition: (a) DMPC; (b) DMPC/DPPG (molar ratio 9:1) were used. DMPC was chosen for the study as a synthetic analog of phosphatidylcholine, the main component of the outer lipid membrane of living cells. DPPG (anionic dipalmitoylphosphatidylglycerol) was used to prepare a negatively charged membrane simulating the charge of on a living cell.

**Figure 2:**
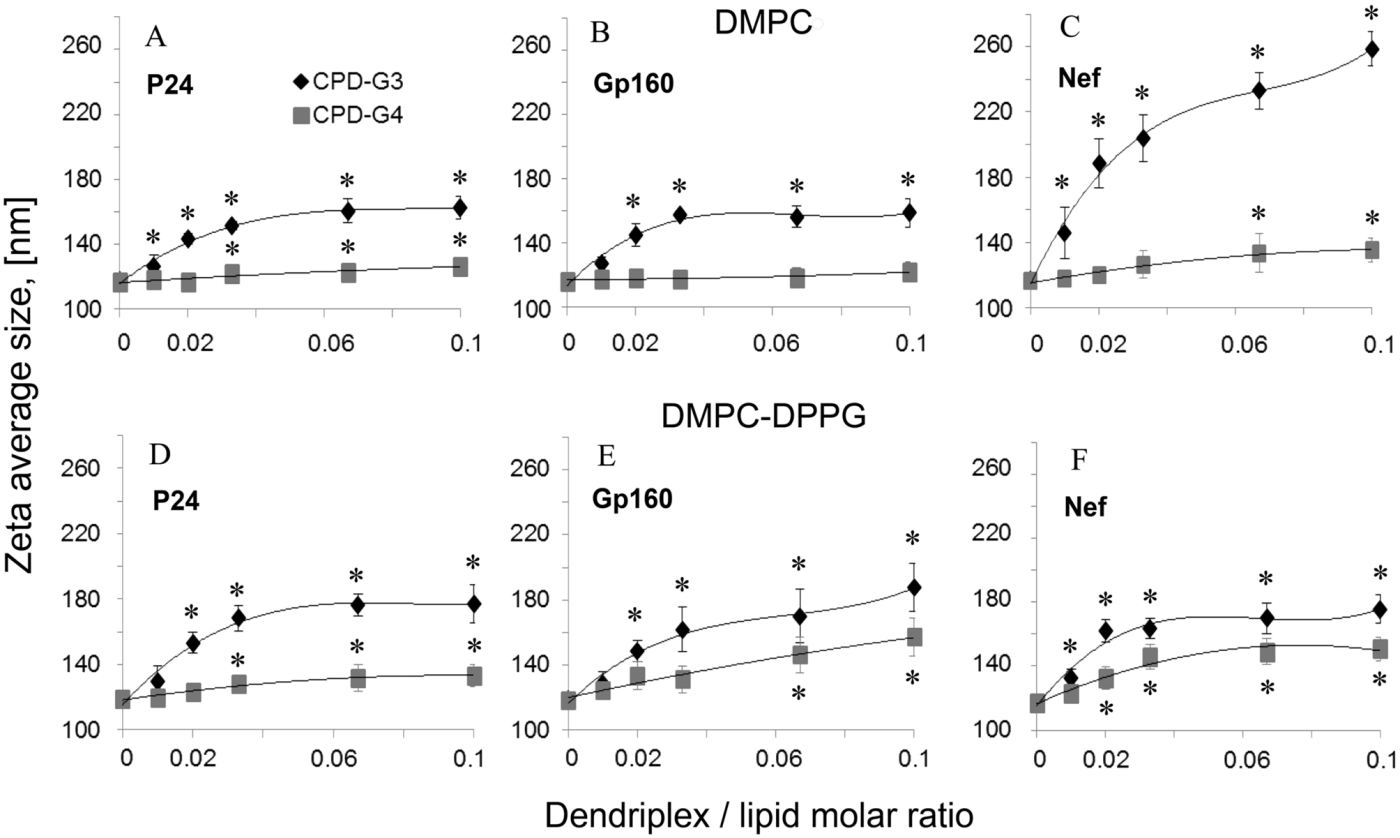
Average size of liposomes upon addition of cationic phosphorus dendrimers/HIV peptide complexes for each combination of peptides and dendrimers (**CPD-G3** or **CPD-G4**): (**A**) DMPC + P24 + Dendrimers; (**B**) DMPC + Gp160 + Dendrimers; (**C**) DMPC + Nef + Dendrimers; (**D**) DMPC–DPPG + P24 + Dendrimers; (**E**) DMPC–DPPG + Gp160 + Dendrimers; (**F**) DMPC–DPPG + Nef + Dendrimers. Results are mean ± S.D., n = 6. *Statistically significant differences in comparison to the control (liposomes) (*p < 0.05), and statistically significant differences between dendrimers (CPD-G3 and CPD-G4) for all points for DMPC + Nef + D and from molar ratio 0.02 for all other systems.

To characterize the interactions between liposomes and dendriplexes, a mean diameter and zeta potential were measured. Changes in the average size of the vesicles in the presence of different concentrations of dendriplexes are shown in Fig. 2. The hydrodynamic diameters of the DMPC or DMPC/DPPG liposomes were 116.5 ± 2.5 and 118.2 ± 1.8 nm, respectively, which is in good agreement with the diameter of the polycarbonate filter used for liposome preparation. For liposomes composed of DMPC, an increase in mean vesicle diameter occurred only when dendriplexes (**CPD-G3** and peptides) were present in the suspension. DMPC liposome size increased to 162.6 ± 6.8 nm (P24), 159.0 ± 8.9 nm (GP160) and 258.7 ± 10.3 nm (Nef) for complex concentration higher than 0.03. We observed the changes in liposome size for peptide/**CPD G4** dendriplexes (Fig. 2, lower curves) except the DMPC liposome with complex Gp160/**CPD-G4** (Fig. 2B). However, the sizes of nano-systems formed with **CPD-G3** were significantly larger than those formed using **CPD-G4** dendrimers.

The hydrodynamic diameter of DMPC–DPPG liposomes in the presence of the **CPD-G3** dendriplex/lipid molar ratio (0.1:1) increased to 176.9 ± 11.5 nm (P24), 187.6 ± 14.9 nm (Gp160) and 175.7 ± 8.8 nm (Nef). For **CPD-G4** dendrimer, the particle sizes changed to 133.3 ± 6.6 nm (P24), 157.2 ± 11.4 nm (Gp160) and 150.8 ± 7.4 nm (Nef) (Fig. 2).

Such results indicate that **CPD-G3** dendriplexes interacted more strongly than **CPD-G4** ones with both (DMPC, DMPC–DPPG) model membranes.

The size of negatively charged DMPC/DPPG liposomes increased significantly in the presence of the dendriplexes, that suggested formation of larger aggregates of liposome–dendriplexes due to the strong adsorption of positively charged dendriplexes onto negatively charged liposome surfaces.

The physical state of the membrane was influenced by interactions with dendrimers and this effect was dependent on the generation as well as concentration of dendrimers^22^. On the other hand, it was demonstrated that in the case of naked HIV peptides (P24, Nef and Gp160), a negatively charged thin layer is formed on the liposome surface, which does not contribute significantly to the LUV diameter^11^.

Each charged particle in a solution containing ions is surrounded by an electrical double layer of ions and counterions. The potential on this hydrodynamic boundary is the zeta potential; measurement of changes in the potential characterise the nature of the interactions between molecules.

**Figure 3:**
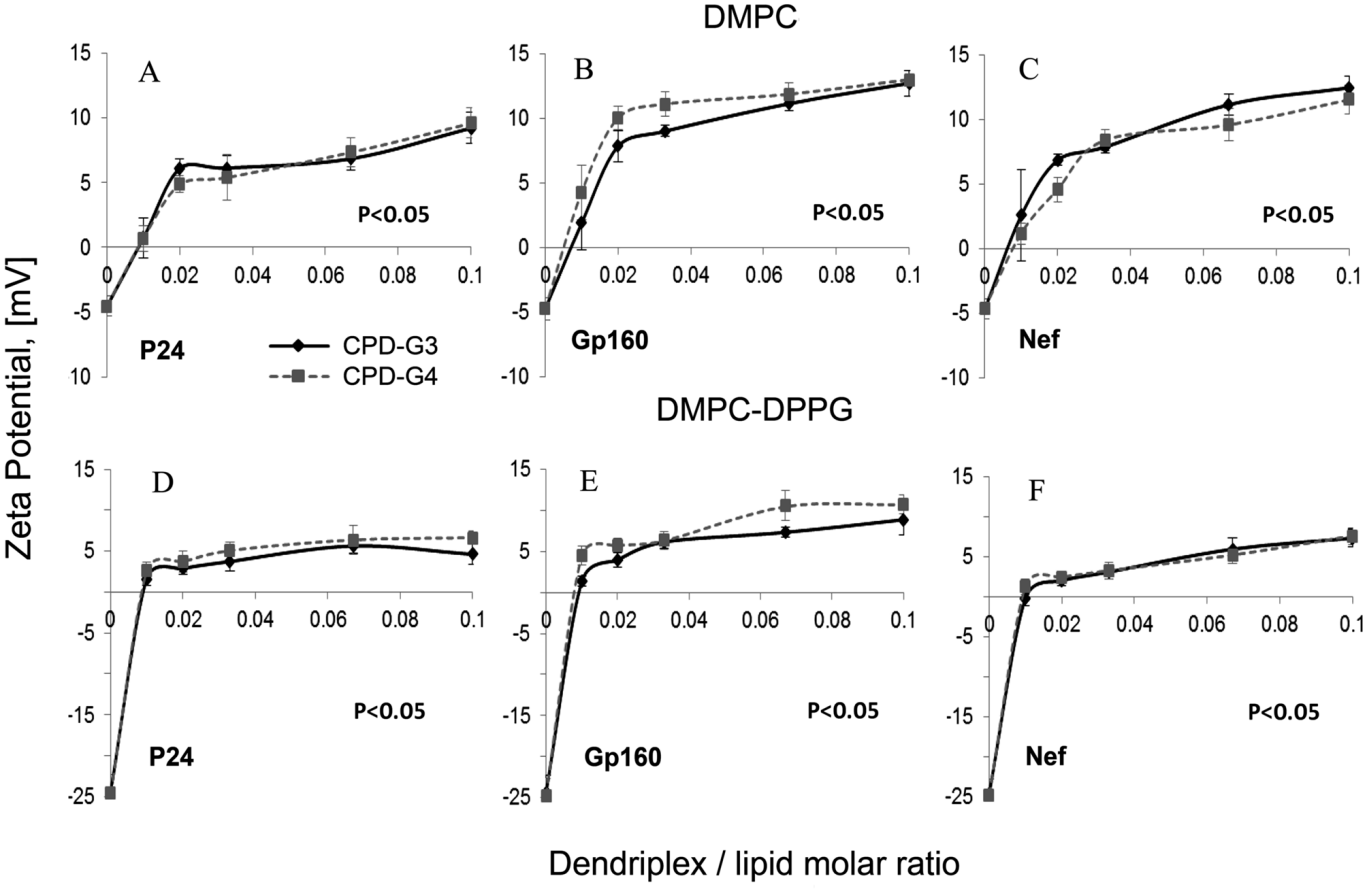
Changes in zeta potential of liposomes upon addition of cationic phosphorus dendrimers/HIV peptides complexes: (**A**) DMPC + P24 + Dendrimers; (**B**) DMPC + Gp160 + Dendrimers; (**C**) DMPC + Nef + Dendrimers; (**D**) DMPC–DPPG + P24 + Dendrimers; (**E**) DMPC–DPPG + Gp160 + Dendrimers; (**F**) DMPC–DPPG + Nef + Dendrimers. Results are mean ± S.D., n = 6. Statistically significant differences in comparison to the control (liposomes without complexes) were *p* < 0.05 in each considered combination.

The effect of phosphorus dendrimers/HIV peptides complexes on zeta potential of liposomes is presented in Fig. 3. The zeta potentials of the control liposomes are − 4.6 ± 0.9 mV and − 24.6 ± 2.1 mV for DMPC, DPMC–DPPG, respectively. DMPC is a zwitterionic lipid that forms membranes with practically a zero surface charge density at physiological pH. This is also indicated by zeta potential for DMPC-LUVs, which were close to zero. Liposomes composed of a mixture of DMPC and DPPG lipid have a different surface charge from the LUVs of DMPC containing only. The mixture of DMPC and DPPG lipid made it possible to build a model membrane with negative surface potential, which better mimics the living cell membrane. Addition of dendriplexes to liposomes significantly increased zeta potential from negative to positive values. Zeta potential values of liposomes after the addition of dendriplexes with **CPD-G3** were not significantly different from those of the dendriplexes containing **CPD-G4** (Fig. 3). Thus the changes in zeta potential resulted from the dendriplexes interaction with liposomes.

Our previous work^11^ presented that HIV-derived peptides (not complexed with dendrimers) decrease zeta potential of DMPC-LUVs, the result of adsorption of negatively charged peptides to a liposome surface. Addition of peptides complexed with carbosilane dendrimers shifted the zeta potential of DMPC and DMPC/DPPG liposomes towards positive values due to the positive surface charge of dendriplexes.

### Transmission electron microscopy (TEM)

**Figure 4:**
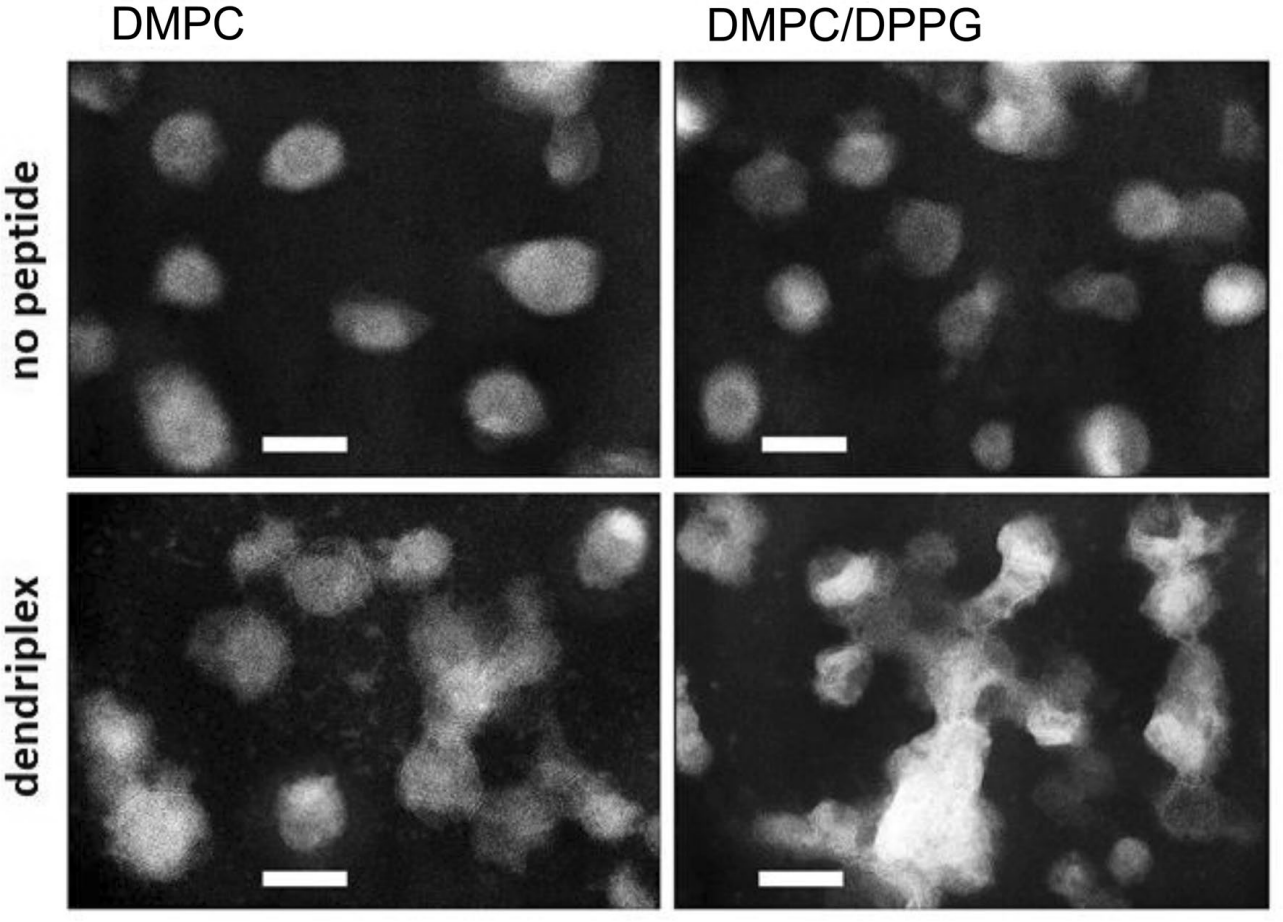
Electron micrographs corresponding to DMPC or DMPC/DPPG lipid vesicles (upper panel) and their mixture with a dendriplex p24/**CPD-G3**, dendriplex/lipid molar ratio 0.04 (lower panel). Dendriplexes were formed in a 10 mM Na-phosphate buffer, pH 7.4 and immediately mixed with liposome suspension. A magnification of ×100,000 was used to examine the samples. Bar = 100 nm. To obtain greater contrast, the color of the microphotographs has been inverted.

TEM images previously confirmed the formation of complexes between **CPD-G3**, **CPD-G4** and HIV-derived peptides^23^. For our TEM investigation we chose only one peptide (P24) and the third-generation dendrimer (**CPD-G3**) for presentation of the morphology of dendriplexes and their complexes with lipid vesicles. The dendriplex was added to the liposomes suspended in 10 mM Na-phosphate buffer at a molar ratio of the 1:25. The morphology of uncomplexed lipid vesicles was compared (top panels Fig. 4) with those complexed with the dendriplexes (bottom panels). The mean average hydrodynamic diameter of the liposomes (116.5 nm and 118.2 nm for DMPC and DMPC–DPPG, respectively), confirmed that the applied approach for vesicle isolation was correct. DMPC and DMPC/DPPG liposomes incubated with peptide/dendrimer complexes had differences in the shape and morphology compared with untreated liposomes, indicating that dendriplexes interact with both types of liposomes. The complexes between dendriplexes and DMPC/DPPG liposomes were larger than the complexes with DMPC liposomes. Our previous work^11^ observed that size and morphology of liposomes conjugated with dendriplexes formed using carbosilane dendrimers were different than size and shape of untreated liposomes. Such dendriplexes interacted with both types of applied liposomes (DMPC and DMPC/DPPG).

The results of TEM correlate with the data obtained by other techniques, and add further support for the stronger interaction of dendriplexes with the negatively charged lipid membranes, which can be explained by the electrostatic nature of interaction between dendriplexes and the lipid membrane.

### Differential scanning calorimetry

**Figure 5:**
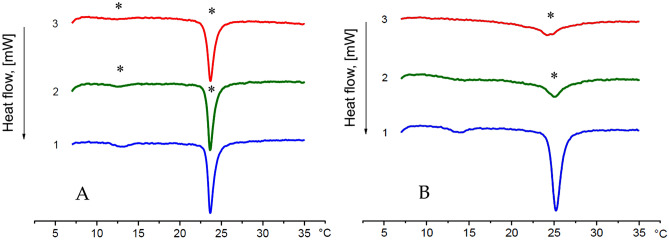
(**A**) DSC heating scans of DMPC membraine in the presence of cationic phosphorus dendrimers G3 complexed with Gp160 HIV-1 peptide (Gp160:G3 1:3, molar). Curve 1 presents the DSC transition of pure DMPC liposomes in absence of dendriplex; 2—in presence of dendriplex at the dendriplex:lipid molar ratio 0.02; 3—dendriplex:lipid molar ratio 0.05. (**B**) DSC heating scans of DMPC:DPPG (9:1, w/w) membrane in the presence of cationic phosphorus dendriplex G3 containing Gp160 (Gp160:G3 1:3, molar). Curve 1 presents the DSC transition of pure DMPC:DPPG liposomes in absence of dendriplex; 2—in presence of dendriplex at the dendriplex:lipid molar ratio 0.02; 3—dendriplex:lipid molar ratio 0.05. Transition peaks with statistically significant (*p < 0.05) areas compered to peaks of membranes without dendriplex treatment.

The DSC heating profiles (Fig. 5A) of zwitterionic DMPC bilayer in phosphorous 10 mM buffer (pH = 7.4) for pure lipid (curve 1) and lipid suspension with increasing concentrations of Gp160:**CPD-G3** dendriplex (curves 2 and 3) exhibit two endothermic transitions upon heating: the pre-transition and the main transition.

Table 1 present the thermotropic parameters of the peaks. The changes of thermotropic parameters of pretransition peak (T_m_ ~ 12.6 °C) shows the effect of dendriplex on the surface structure of DMPC lipid membrane. The change of main transition peak (T_m_ ~ 23.7 °C), reflects the interaction of dendriplex with hydrophobic part of the bilayer. Increased concentrations of dendriplexes, caused their stronger interaction with the bilayer surface. This effect is confirmed by the alterations of the parameters of pretransition peak. The onset temperatures (T_onset_), have been decreased from 11 to 10.2 °C, while the peak area was reduced up to 58% (ΔH) and peak has been broaden up to 70% (T_1/2_).

**Table 1:** DSC parameters of Gp160/CPD-G3 dendriplexes interaction with (A)—DMPC (5 mg/ml) and (B)—DMPC:DPPG (9:1 w/w, 5 mg/ml) lipid membranes.

Dendriplex:lipid molar ratio	T_onset_ [°C]	T_m_ [°C]	ΔH [kJ/mol]	T_1/2_ [°C]
**A**				
DMPC, pretransition peak				
0.00	11.00 ± 0.31	12.65 ± 0.35	1.90 ± 0.08	1.91 ± 0.09
0.02	10.86 ± 0.55	12.58 ± 0.16	1.53 ± 0.09*	2.15 ± 0.06
0.05	10.17 ± 0.15	12.07 ± 0.20	0.80 ± 0.09*	3.25 ± 0.34*
DMPC, main transition peak				
0.00	23.15 ± 0.01	23.67 ± 0.01	19.02 ± 0.31	0.80 ± 0.01
0.02	23.1 ± 0.01*	23.65 ± 0.01	17.44 ± 0.30*	0.79 ± 0.01
0.05	23.02 ± 0.02*	23.69 ± 0.01	17.58 ± 0.14*	0.90 ± 0.01*
**B**				
DMPC/DPPG, pretransition peak				
0.00	12.58 ± 0.04	13.97 ± 0.17	2.00 ± 0.12	2.09 ± 0.14
0.02	12.49 ± 0.55	13.90 ± 0.60	1.81 ± 0.08	3.76 ± 0.51*
0.05	–	–	–	–
DMPC/DPPG main transition peak				
0.00	24.32 ± 0.01	25.22 ± 0.01	18.90 ± 0.29	1.17 ± 0.02
0.02	23.49 ± 0.20*	25.07 ± 0.06	9.82 ± 0.57*	2.42 ± 0.58
0.05	22.90 ± 0.22*	24.42 ± 0.27*	14.89 ± 0.66*	3.27 ± 0.39

Calorimetric parameters: T_onset_—temperature at which the thermal effect starts; T_m_—temperature at which heat capacity at constant pressure is maximum; ΔT_1/2_—half width of the peak transition; ΔH—transition enthalpy. Errors express as SEM, n = 3.

*Statistically significant difference at *p < 0.05 compared to lipid membranes without dendriplex treatment.

The interaction of Gp160:**CPD-G3** dendriplex with interior of DMPC bilayer is weaker in comparison with surface interaction, as can be seen from main transition parameters: decrease in onset temperature (T_onset_) from 23.15 to 23.02 °C, slightly broadening the peak (increase in T1/2 by 12.5%) and little reduction in the peak area (decrease in ΔH by 7.6%).

DSC studies (Fig. 5B) of the impact of increasing Gp160:**CPD-G3** dendriplex concentration on the structure of anionic DMPC–DPPG (9:1, w/w) membrane also confirmed two kinds of transititions. Thermotropic parameters for pretransition and main transition (Table 1) reveal significant interactions of studied dendriplex with DMPC–DPPG membrane. The highest applied dendriplex concentration (at dendriplex to lipid molar ratio 0.05/1) is enough to abolish the pretransition peak (T_m_ ~ 14 °C). Dendriplex also affected the hydrophobic region of bilayer structure, as can be seen from decreasing in onset temperature T_onset_ (from 24.3 to 22.9 °C) and decreasing in main transition temperature T_m_ (from 25.2 to 24.4 °C), distinct broadening of the peak (increase of T_1/2_ by 180%) and reduction in the peak area (decrease of ΔH by 21%) for main transition.

Comparing Gp160:**CPD-G3** dendriplex impact on both studied membranes reveals that cationic Gp160:**CPD-G3** dendriplex interacts stronger with anionic DMPC–DPPG membrane then with the zwittereionic DMPC one. The increase of electrostatic attraction between cationic dendriplex and anionic DMPC–DPPG membrane enables not only the interactions with the surface of the membrane (abolishing of pretransition for DMPC–DPPG and area reduction for DMPC) but also intensifies the dendriplex impact on hydrophobic interior of lipid bilayer (broadening of main transition T_1/2_ by 180% for DMPC–DPPG in comparison with 12.5% for DMPC and reduction in main transition enthalpy ΔH by 21% for DMPC–DPPG and by 7.6% for DMPC).

Interaction of **CPD**s with model lipid membranes indicated that **CPD**s interacted with DMPC membranes through random contacts, whereas the presence of a negative charge in the lipid membrane led to dendrimers interaction with the hydrophilic part of the membranes^24^.

### Fluorescence anisotropy

Fluorescence spectroscopy was applied to evaluate the effect of dendrimer/peptide complexes on the membrane fluidity. For this purpose, two fluorescent probes, TMA-DPH and DPH, were used. These probes were selected due their different localization in the belayer. The TMA-DPH is located in the polar region of the lipid membrane, while DPH in the hydrophobic part of the membrane^25,26^.

**Figure 6:**
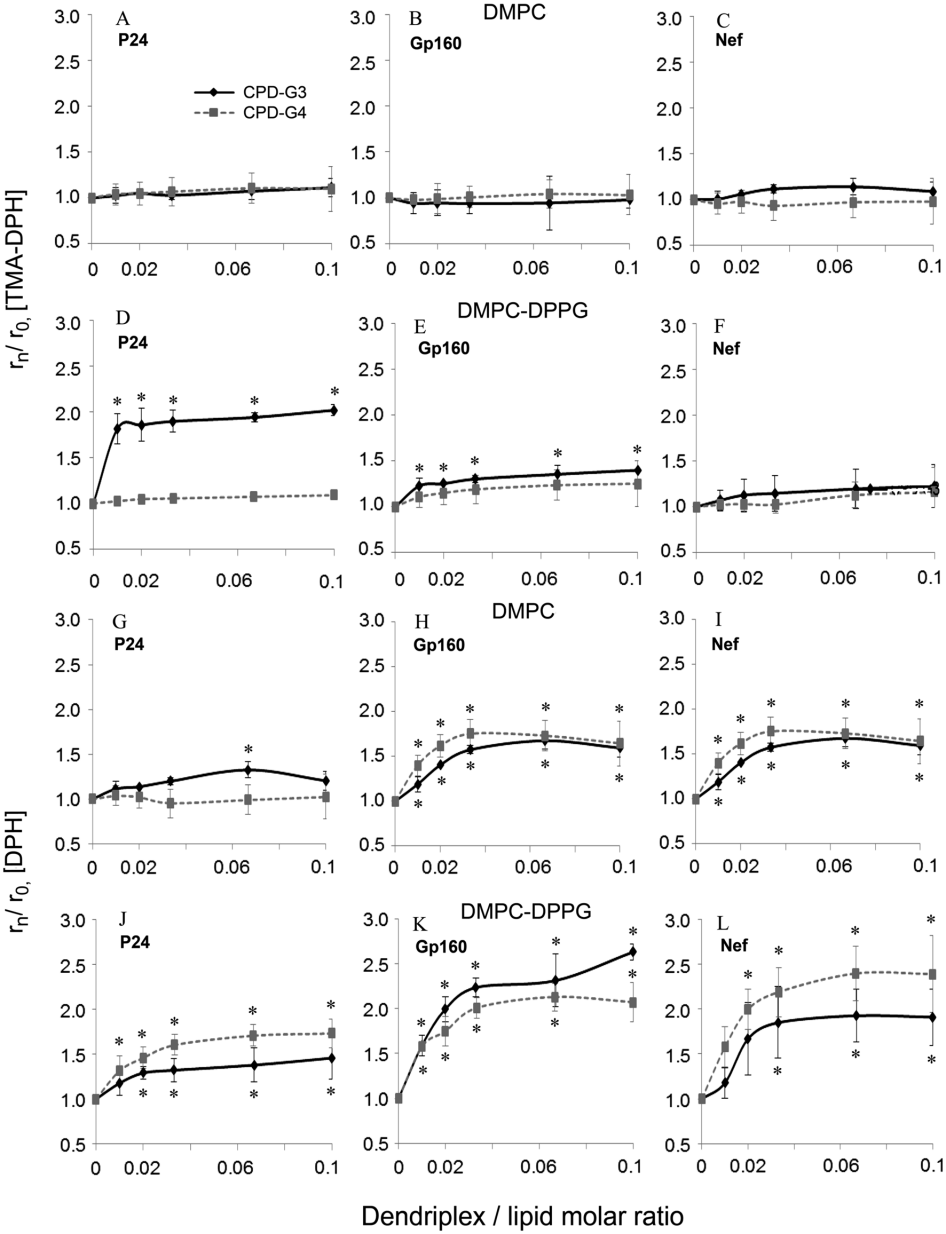
Fluorescent anisotropy of TMA-DPH probes in liposomal membranes containing DMPC or DMPC/DPPG (9:1) w/w phospholipids in the presence of cationic phosphorus dendrimers/ HIV peptides complexes: (**A**) DMPC + P24 + Dendrimers; (**B**) DMPC + Gp160 + Dendrimers; (**C**) DMPC + Nef + Dendrimers; (**D**) DMPC–DPPG + P24 + Dendrimers; (**E**) DMPC–DPPG + Gp160 + Dendrimers; (**F**) DMPC–DPPG + Nef + Dendrimers and fluorescent anisotropy of DPH probes in liposomal membranes: (**G**) DMPC + P24 + Dendrimers; (**H**) DMPC + Gp160 + Dendrimers; (**I**) DMPC + Nef + Dendrimers; (**J**) DMPC–DPPG + P24 + Dendrimers; (**K**) DMPC–DPPG + Gp160 + Dendrimers; (**L**) DMPC–DPPG + Nef + Dendrimers. The molar ratio of the probe to the lipid = 1:100. The results are mean ± S.D., n = 6. *Statistically significant differences in comparison to the control (*p < 0.05) and between dendrimers (CPD-G3 and CPD-G4) ^#^*p* < 0.05.

The influence of dendriplexes on the fluorescence anisotropy of liposome is shown in Fig. 6. Results show the fluorescence anisotropy of probes located in liposomes at the presence or absence of studied dendriplexes. The addition of CPD-G3/P24 and CPD-G3/Gp160 complexes to DMPC–DPPG liposomes significantly increased the value of TMA-DPH fluorescence anisotropy, which means that they stiffened the membrane. In contrast, no significant changes in the fluorescence anisotropy of TMA-DPH with DMPC liposomes were observed, indicating that the dendriplexes did not alter the membrane fluidity in the polar head region.

No significant changes in DPH fluorescence anisotropy were observed in DMPC liposomes exposed to **CPD-G4**/P24 complexes, but the values of DPH fluorescence anisotropy increased for the other dendriplexes. All the dendriplexes also increased the fluorescence anisotropy in DMPC–DPPG LUVs. In our previous work^11^ we observed that increased concentration of dendriplexes formed using carbosilane dendrimers in the suspension of DMPC/DPPG liposomes significantly increased the TMA-DPH fluorescence anisotropy. The effect was stronger for the dendriplexes based on CBD-CS dendrimers in comparison with CBD-OS dendrimers. Such effect on the fluorescence anisotropy of DPH probe was not uniform, showing a weaker effect of dendriplexes on the hydrophobic domain of the membrane.

Naked peptides do not affect membrane fluorescence anisotropy either for neutral or negatively charged liposomes^11^. Wrobel et al.^27^ showed that the presence of phosphorus-containing dendrimers in DMPC or DPPC liposomal suspension at temperatures above the main transition point for both lipids (DMPC—37 °C; DPPC—50 °C) led to significant changes in the fluorescence anisotropy of either of the probes used. Increase fluorescence anisotropy can be interpreted as greater rigidity of the liposomal membranes, probably due to dendrimers moving into the liposome bilayer. No significant changes in either DPH or TMA-DPH anisotropy for DMPC liposomes were found for lipid to dendrimer interactions below the main transition temperature^11^. For DPPC liposomes, the results suggest a lack of interaction between phosphorus-containing dendrimers with vesicles at a temperature below the transition point. There were only weak interactions between lipids and dendrimers.

### Surface pressure on lipid monolayers

The mechanisms of interactions between the lipid membrane and HIV-peptide/dendrimer complexes were examined by the monolayer technique, which is based on surface pressure-area isotherm analysis or measurement of the changes of surface pressure in a steady-state condition^27^. In the latter case, the lipid monolayer is formed to reach a predefined surface pressure; after establishing steady-state conditions, the kinetics of surface pressure changes is measured after adding the dendriplexes. The monolayers were composed of DMPC/DPPG mixture (9:1) w/w at the air–water interface, with Na-phosphate buffer (10 mM, pH 7.4) being used as a sub-phase. The surface pressure of monolayers was set at 30 mN/m to correspond with the condensed state of the membrane. Adding anionic lipids simulated the natural charge of cell membranes. We had previously found that changes in the surface pressure of monolayers composed of DMPC and DMPC/DPPG under the influence of HIV-derived peptides are not significant^11^.

**Figure 7:**
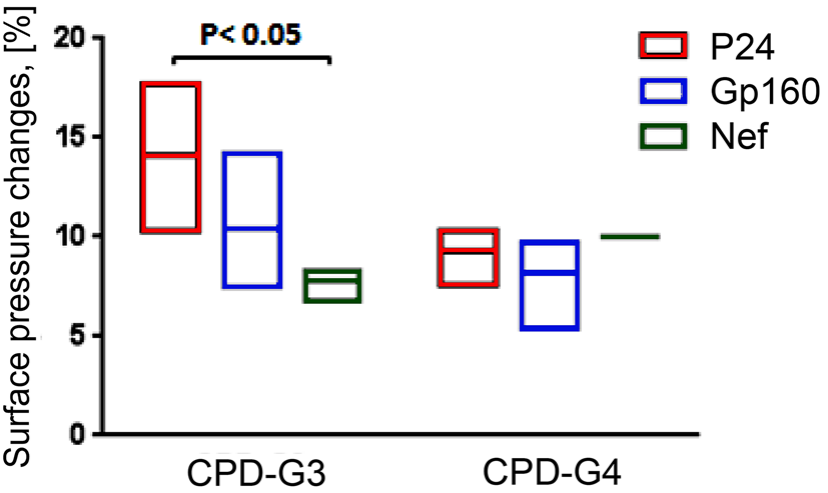
Changes in the surface pressure of monolayers formed by DMPC–DPPG lipid systems following addition of dendriplexes into the buffer subphase onto which the lipid monolayer with initial surface pressure of 30 mN/m was formed. Results represent mean ± S.D. n = 3. Statistically significant differences in comparison to the control (*p* < 0.05).

Furthermore, the results indicated that analyzed dendriplexes interact with DMPC/DPPG lipid monolayers. Changes in DMPC/DPPG monolayer surface pressure in the presence of dendriplexes are shown in Fig. 7. Dendriplexes contained CPD-G3 dendrimer and P24 or Gp160 peptides influenced the surface pressure stronger than dendriplexes contained CPD-G4. At the presence of CPD-G4/Nef complex the interior of the liposomal membranes became more rigid in comparison with untreated membranes. Carbosilane dendrimers increase the surface pressure of lipids; dendriplexes formed using CBD-OS (contained carbon silicon bonds) dendrimers induced more pronounced changes than denriplexes with CBD-Cs (oxygen silicon bonds)ones^11^.

Figure 7 shows that changes in the surface pressure of monolayers for **CPD-G3** declines whereas that for **CPD-G4** is stable, which clearly indicates the presence of an interaction. Thus, the low P value alone can be interpreted as a degree of evidence (in the framework of Fisher's approach^28^) that the difference between peptides was not consistent for each dendrimer type. In other words, the magnitude of the effect of peptide on surface pressure was dependent on the dendrimer generation. In graph (Fig. 7) we could see differences in the effect of peptides complexed with **CPD-G3** with peptide p24 as the one that most influenced the change in surface pressure, whereas the complex of **CPD-G3** with peptide Nef had smaller effect, as with all the complexes with **CPD-G4**.

## Conclusions

The results suggest that HIV-peptide dendrimer complexes can interact with both hydrophobic and hydrophilic parts of lipid membrane. Interactions between dendriplexes and DMPC–DPPG liposomes were stronger than with liposomes composed of DMPC zwitterionic lipids. The nature of interaction probably differs because positively charged dendriplexes interact with the negatively charged membrane by electrostatic interactions. The interaction of neutral liposomes with dendriplexes is due to disturbances in the hydrophobic domain of the membrane. The data collected on monolayer surface, as well as from membrane fluidity, zeta potential, DSC and DLS measurements, provide a basis for the optimization and potential use of dendrimeric nanosystems as carriers for HIV-1 peptides.

The results of this study provide important information about interplaying of antigens (HIV-peptides) with delivery vehicles (dendrimers) and their interaction with lipid membranes. Presented data can be useful for the design of antiviral vaccine systems targeted against HIV and other viruses and while applying other antigens.

## Methods and material

### Dendrimers

**Figure 8:**
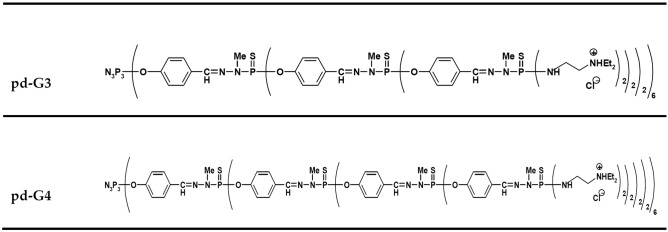
Structure of phosphorus-containing dendrimers of generations 3 and 4.

The third and fourth generation of phosphorus dendrimers (**CPD**—structure shown in Fig. 8 with surface cationic end groups were used. **CPD-G3**, C_624_H_1104_N_183_Cl_48_O_42_P_45_S_42_ (generation 3, 48 surface cationic end groups, MW: 16,280 g/mol; diameter: 4.1 nm) and **CPD-G4**, C_1296_H_2256_N_375_Cl_96_O_90_P_93_S_90_ (generation 4, 96 surface cationic end groups, MW: 33,702 g/mol; diameter: 5 nm). Dendrimers were synthesized in the Laboratoire de Chimie de Coordination du CNRS. The main characteristics and synthesis of **CPD**s have been described by Caminade and Majoral^15^.

### HIV-derived peptides

Three different HIV-derived peptides were used: (i) A peptide derived from the envelope Gp160 sequence, HIVHXB2 location Gp160 (634e648): NH-EIDNYTNTIYTLLEE-COOH, length of 15 amino acids, charge (− 4);

(ii) A peptide derived from the Gag-P24 sequence, HIV-HXB2 location P24 (71e80): NH-DTINEEAAEW-COOH, length 10 amino acids, charge (− 4);

(iii) A peptide derived from the Nef sequence, HIV-HXB2 location Nef (172–191): NHGMDDPEREV-LEWRFDSRLAFCOOH, length 20 amino acids, charge (− 3).

All HIV-derived peptides were prepared by Eurogentec S.A. (Belgium).

### Phospholipids used for liposomes and monolayers preparation

The phospholipids used—1,2-dimyristoyl-sn-glycero-3-phosphatidylcholine (DMPC) and 1,2-dipalmitoyl-sn-glycero-3-phosphatidylglycerol (DPPG)—were purchased from Avanti Polar Lipids Inc. (USA). All other reagents of analytical grade were from Sigma-Aldrich (USA) or POCH (Gliwice, Poland). Solutions were made with water purified with the Milli-Q system (Millipore Corporation, Billerica, MA, USA).

### Preparation of peptide–dendrimer complexes (dendriplexes)

Peptide–dendrimer complexes were formed by adding dendrimers to peptide solutions at a molar ratio of 1:3 (peptide:dendrimer). **CPD** dendrimers and HIV-derived peptides were dissolved in a 10 mM Na-phosphate buffer pH 7.4. The mixture was vortexed and incubated for 10 min at room temperature (~ 20 °C). Selection of dendrimers and the peptide molar ratio for studying the interaction of dendriplexes with liposomes was based on the work by Ciepluch et al.^23^. The authors showed that both generations of dendrimers (G3 and G4) form stable complexes with all kinds of studied HIV-peptides at a 1:2–3 molar ratio (peptide:dendrimer).

### Preparation of liposomes

To prepare the liposomes (LUVs), appropriate amounts of DMPC or DMPC/DPPG mixtures (9:1 w/w) were dissolved in chloroform and the solvent evaporated under vacuum. The dry lipid film formed was hydrated with 10 mM Na-phosphate buffer (pH 7.4) to a lipid concentration of 5 mg/ml, which was extruded 15–17 times through Millipore polycarbonate filters (100 nm pore size) using an Avanti extruder (Avanti Polar Lipids, USA) to obtain LUVs. The temperature during extrusion was kept at approx. 37 °C, i.e. well above the main phase-transition temperature of the lipids (~ 24 °C).

### Measurement of particles size

The particle size of liposomes in the absence/presence of dendriplexes was measured by a dynamic light scattering (DLS) technique using a Zetasizer Nano-ZS (Malvern Instruments, UK). Samples in 10 mM Na-phosphate buffer, pH 7.4, filtered through 0.22 μm filters, were placed in plastic Malvern cells (DTS0012) and measured at 25 °C. The refraction factor was assumed to be 1.33 at a detection angle of 90° at a wavelength of 633 nm. Malvern software was used to analyze the data.

### Measurement of zeta potential

The particle charge was measured with a Zetasizer Nano-ZS. The electrophoretic mobility of the samples in an applied electric field was measured in Malvern capillary plastic cells (DTS1070). The zeta potential was calculated directly from the Helmholtz–Smoluchowski equation with the Malvern software^29,30^. HIV-derived peptides alone or complexed with **CPD** dendrimers were added to 1 ml liposome suspension to obtain dendriplex/lipid molar ratios of 1:100, 1:50, 1:30, 1:15, and 1:10. Samples were prepared in 10 mM Na-phosphate buffer pH 7.4, filtered as above, and measured at 25 °C. Zeta potentials were the average of 9–11 measurements.

### Differential scanning calorimetry

The impact of Gp160:**CPD-G3** dendriplex (Gp160 protein to G3 dendrimer molar ratio 1:3) on the structure of zwitterionic membrane composed from DMPC as well as anionic membrane composed from DMPC and DPPG (9/1, w/w) was investigated by differential scanning calorimetry (Setaram DSC III microcalorimeter). Investigated lipids were initially dissolved in chloroform and subsequently dried at 38 °C and resuspended in 10 mM phosphorous buffer (pH = 7.4). The final concentrations of lipids were the same in all studied samples: 5 mg/ml (7.38 mM) DMPC for zwitterionic membrane and 4.5 mg/ml DMPC (6.64 mM) and 0.5 mg/ml DPPG (0.67 mM) for anionic DMPC– DPPG membrane (9/1, w/w). DSC measurements were carried out for zwitterionic DMPC membrane as well as anionic DMPC–DPPG membrane with the increasing Gp160:**CPD-G3** dendriplex concentration. The molar ratio of dendriplex/lipid in studied mixtures was subsequently 0.02/1 (0.15 mM Gp160, 0.45 mM **CPD-G3**) and 0.05/1 (0.37 mM Gp160, 1.11 mM **CPD-G3**). Each studied sample (440 μl) was scanned 3 times from 5 to 40 °C with scan rate 0.5 °C/min. From each recorded DSC curve the reference scan (440 μl 10 mM phosphorous buffer with pH = 7.4) was subtracted. Enthalpies of transitions and characteristic temperatures were calculated using Setaram software.

### Steady-state fluorescence spectroscopy

Steady-state fluorescence anisotropy measurements were carried out with a Perkin–Elmer (U.K.) LS-50B spectrofluorimeter. To monitor membrane fluidity of a bilayer, two fluorescent probes were used: DPH (l,6-diphenyl-l,3,5-hexatriene), an apolar molecule, is incorporated into the hydrophobic region of the liposome bilayer, whereas TMA-DPH (1-(4-(trimethylamino)phenyl)-6-phenylhexa-1,3,5-triene) probe located near the lipid head groups. The molar ratio of the phospholipid to the fluorescent probe was 100:1. Excitation was at 348 nm and emission at 426 nm in the analysis of the degree of probe mobility in the bilayer^31–33^. For both probes the slits widths used were 5 nm and 3 nm for excitation and emission monochromators, respectively. The measurements were performed at 25 °C.

### Monolayers method

A Langmuir–Blodgett technique was used to monitor the interactions between dendriplexes and lipid monolayers composed of a mixture of DMPC and DPPG. A small circular teflon cell (10 ml) was used. Appropriate amounts of lipid dissolved in chloroform at 0.3 mg/ml were added to the surface of the 10 mM Na-phosphate buffer to reach a surface pressure of close to 30 mN/m, corresponding to the condensed state of the monolayer. Wilhelmy’s method was applied to measure the surface pressure using a PS4 sensor (NIMA Technology, UK)^34^. When the surface pressure had stabilized, peptide/dendrimer complexes were added at 0.25 μM to the water sub-phase (i.e. under the monolayer) by a Hamilton syringe through a special channel, and the surface pressure was recorded until the steady state had been reached.

### Transmission electron microscopy (TEM)

The morphology of liposomes under the presence of peptide/dendrimer complexes was examined by transmission electron microscopy JEOL-10 (Japan). Dendriplexes were added to liposomes suspended in the 10 mM Na-phosphate buffer at a molar ratio of 1:25 (dendriplex:lipid). The resultant complexes were placed on the carbon surface of a 200-mesh copper grid (Ted Pella, Inc., USA) for 10 min and drained with blotting paper. The samples were stained with 2% (w/v) uranyl acetate for 2 min and dried. A magnification of 50,000–100,000× proved best for examining liposomes interacting with peptide/dendrimer complexes.

### Statistical analysis

All experiments (except experiments on monolayers) were run at least three times. Values are given as the mean ± standard deviation of at least four independent experiments. One-way Analysis of Variance (one-way ANOVA) followed by Tuckey’s analysis has been applied.
